# A grounded theory of regaining normalcy and reintegration of women with obstetric fistula in Kenya

**DOI:** 10.1186/s12978-019-0692-y

**Published:** 2019-03-08

**Authors:** Anne M. Khisa, Isaac K. Nyamongo, Grace M. Omoni, Rachel F. Spitzer

**Affiliations:** 1 University of Nairobi, School of Nursing Sciences, P.O. Box 30197-00100, Nairobi, Kenya; 2 African Population and Health Research Centre, P.O. Box 10787-00100, Nairobi, Kenya; 3 Division of Cooperative Development, Research and Innovation, The Cooperative University of Kenya, P.O. Box 24814-00502, Nairobi, Kenya; 4 University of Nairobi, Institute of Anthropology, Gender and African Studies, P.O. Box 30197-00100, Nairobi, Kenya; 5 University of Nairobi, School of Nursing Sciences, P.O. Box 30197-00100, Nairobi, Kenya; 6 University of Toronto, Dalla Lana School of Public Health, 155 College St. ON M57 3M7, Toronto, Canada

**Keywords:** Reintegration, Regaining normalcy, Kenya, Obstetric fistula, Narratives, Grounded theory

## Abstract

**Background:**

Obstetric fistula is a reproductive health problem causing immense suffering to 1% of women in Kenya that is formed as sequelae of prolonged obstructed labour. It is a chronic illness that disrupts women lives, causing stigma and isolation. Fistula illness often introduces a crisis in women’s life begetting feelings of shame and serious disruption to their social, psychological, physical and economic lives, in addition to dealing with moral and hygiene challenges. Currently, women undergo free of charge surgery at vesicovaginal fistula (VVF) camps held in national referral hospitals and dedicated fistula centres generating a significant pool of women who have undergone surgery and are ready to regain normal lives.

**Objective:**

The purpose of this study was to explore experiences of women immersing back into communities and their return to normalcy after surgery in three VVF repair centres in Kenya. We set out to answer the question: what strategies improve obstetric fistula patients’ reintegration process?

**Methods:**

We used grounded theory methodology to capture the reintegration and regaining normalcy experiences of women after surgery. Narrative interviews were held with 60 women during community follow-up visits in their homes after 6–19 months postoperatively. Grounded theory processes of theoretical sampling, repeated measurement; constant comparative coding in three stage open, axial and selective coding; memoing, reflexivity and positionality were applied. Emergent themes helped generate a grounded theory of reintegration and regaining normalcy for fistula patients.

**Results:**

To regain normal healthy lives, women respond to fistula illness by seeking surgery.. After surgery, four possible outcomes of the reintegration process present; reintegration fully or partially back into their previous communities, not reintegrated or newly integrating away from previous social and family settings. The reintegration statuses point to the diversity outcomes of care for fistula patients and the necessity of tailoring treatment programs to cater for individual patient needs.

**Conclusion:**

The emerging substantive theory on the process of reintegration and regaining normalcy for fistula patients is presented. The study findings have implications for fistula care, training and policy regarding women’s health, suggesting a model of care that encompasses physical, social, economic and psychological aspects of care after surgery and discharge.

## Plain English summary

One percent of Kenyan women suffer from vaginal fistula, an illness developed during difficult childbirth. The illness causes incontinence of urine and faeces, and is mentally distressing. Further, women who suffer vaginal fistula are social isolated and stigmatised, and do not participate in education or economic activities. Being poor, the women are treated by surgery that is free of charge by various partnerships between public and private hospitals, university faculty and Non-governmental organisations.

We conducted a study to explore the experiences of 60 women who had undergone surgery from three hospitals. In the interviews, we focused on how they were able to settle back in the same communities that had previously shunned them. In addition we established their needs during the healing process for both physical and psychological illness. We used qualitative research to generate a theory that explains the process of reintegration for fistula patients after surgery. We propose strategies that can improve the reintegration process, which are hypotheses emerging from the reintegration theory.

In our theory, we point out that the assumption that women will automatically heal after surgery is not true. We found that they may heal fully, partially or not at all depending on their needs and the context after surgery. There is therefore need for a special follow up of women with fistula after fistula surgery. This follow up care should include both counselling and psychological support needs, medical and physical care, social and family support and economic recovery strategies as part of wholesome care for women with fistula.

## Introduction

Obstetric fistula is prevalent in 1% of women in Kenya [1]. The prevalence of symptoms of vaginal fistula is 1 per 1000 women of reproductive age in 19 Sub-Saharan Africa countries [2]. Women suffering obstetric fistula often experience a stigmatised illness for decades, enduring physical and psychological symptoms in addition to social isolation [3]. The illness disrupts their normal lives as women and wives in the society. Surgical treatment of women suffering from obstetric fistula has gained momentum in Kenya, with an estimate 1200 women treated annually in free one week camps in a few public hospitals and one dedicated fistula repair centre. The focus is on regaining continence and some form of psychological counselling [4]. The patients are discharged with instructions to avoid strenuous work; abstain from sexual relations and eat a balanced diet and exercise. There is one scheduled follow-up visit to hospital at six weeks after surgery. However, surgery treats the physical urine and / or faecal incontinence, setting them off on a path of recovery [5]. There remain many aspects of healing, namely, fertility treatment, resumption of reproductive capacity, healing the psychological trauma and social acceptance back into their communities that ought to result from the treatment and care processes. A preliminary exploratory study showed that patients may need greater support after surgery and discharge than previously thought [6].

Our paper thus offers an understanding of the process of regaining normal lives and reintegration into communities by fistula patients in Kenya, filling an important knowledge gap in fistula care literature. Further, we advance a theory of reintegration concerning one of the most stigmatised reproductive health illnesses. Here, we use the term patients because our study participants had undergone surgery, and upon discharge, they are expected to return after six weeks for a follow up visit. Fistula care is therefore not only during admission in hospital but in the subsequent period. It is not clear whether women still perceive themselves as patients after discharge. Although this line of questioning would be an interesting find, it was beyond the scope of this study.The World Health Organisation (WHO) recommends that individual fistula programs ought to cater for reintegration of fistula patients. Nevertheless, they don’t explicitly recommend how reintegration programs may be designed [7]. Concerning reintegration of fistula patients, several authors have at least reported on surgical outcomes after surgery [8] fistula recurrence and pregnancy outcomes after fistula repair [9]. A project in Uganda developed and an instrument to measure post-surgical reintegration of fistula patients [10, 11]. However, no known study has proposed a full range of interventions relevant to reintegration of fistula patients as our study.

This study was conducted over two phases in 19 months with women who had undergone obstetric fistula surgery. For practical purposes and space limitations, the health seeking behaviours of fistula patients [12] and narratives of the labour that caused the fistula illness [13] were published in separate articles. In this paper, we report on findings of the second phase of the study whose focus was on reintegration of patients after fistula surgery. The key question which we sought to answer was; what are the reintegration experiences and needs of obstetric fistula patients in Kenya? We used grounded theory methodology to answer the research question.

## Materials and methods

### Sampling

At the time of the study, there were varied media campaigns to get free surgery or fistula patients and in two of the three centres, patients had been recruited from such annual fistula camps for the first phase of the study. All patients in our study had undergone surgery free of charge though annual camps or at the all year fistula repair centre. The follow up interviews were conducted mostly in rural areas, however, a few participants lived in urban informal settlements.

We used purposive sampling to recruit participants into the study, basing on three main criteria. First, fistula patients who had developed vaginal fistula from labour and childbirth; secondly, those who had undergone free corrective surgery at one of three hospital sites and third, those who were willing to be followed up by the research team in their communities for periods exceeding 6 months post operatively and voluntarily gave informed consent to participate. In keeping to how large a grounded theory GT study can be, the research drew on the examples of previous work [14].

### Recruitment

There were three women who declined to participate in second phase. However, it is important to clarify that we did not have participants drop out of the study, since we used purposive sampling and stopped seeking participants once we reached data and thematic saturation. We are therefore not able to comment on whether a lot more women would have declined had we continued with the study. Notably, our participants remained available to discuss their health even at 19 months after surgery. We particularly did not experience any follow up activity jeopardizing the reintegration process, perhaps because we took ethical measures of informed consent and protection of study participants.

This section describes the characteristics of women whose narratives we have presented. The percentages and proportions used are appropriate and serve a descriptive purpose only, given that the sample was fully purposive but should not be used for statistical inference. We recruited (60) women and girls aged 17 to 62 (mean 33.2) years into the study. All the women had developed a vaginal fistula during labour and childbirth, and had obtained surgery free of charge at one of the three sites namely Kisii Level 5 Hospital, Gynocare Fistula Centre and Kenyatta National Hospital. On average, the participants had developed obstetric fistula at a much lower age than their current age, mean 21.9 years (range 11–36 years) and had therefore lived with the illness for long, reporting an average of 9.6 years (range 0.17–39.1).

About 13.3% of participants had not obtained any form of formal education, and a further 58.4% only had primary level education. A paltry 6.7% had college level of education, signifying a generally low level of education, literacy and numeracy skills amongst participants. The participants were derived from 30 out of 47 counties in Kenya.

Whilst 26 women (44.1%) of participants had developed fistula illness during their first pregnancy, the majority (55.6%) had developed the illness during the second or higher order delivery. Most participants (96.6%) had visited a hospital or health centre several times before obtaining corrective surgery, either as referrals or first time clients in the fistula centre; and a further 40.7% had obtained multiple surgeries for the ailment, both facts that signify a long and arduous journey to obtaining treatment for their illness. A complete set of descriptive characteristics of our study participants is presented in Table 1 below:

**Table 1:** Characteristics of study Participants

Variable		Frequency (*n*)	Percent (%)
Hospital Treated (*N* = 60)	Gynocare	19	31.7
	Kenyatta National Hospital	18	30.0
	Kisii Level 5 Hospital	23	38.3
Age in Years at time of study (*N* = 60) Mean 33.24 Median 30.0 Mode 28 Range 45 (17–62)	15–19	7	11.9
	20–24	6	10.2
	25–29	15	25.4
	30–34	10	16.9
	35–39	5	8.5
	40–44	5	8.5
	45–49	2	3.4
	50–54	6	10.2
	55–59	2	3.4
	60–64	1	1.7
	Not determined	1	–
Age in years at onset of fistula (*N* = 60) Mean 21.9 Median 22 Mode 19 Range 25 (11–36)	10–14	8	13.6
	15–19	18	30.5
	20–24	13	22.0
	25–29	14	23.7
	30–34	3	5.1
	35–39	3	5.1
	Not determined	1	
Level of Education (*N* = 60)	None	8	13.3
	Primary 1–4	4	6.7
	Primary 5–8	31	51.7
	Secondary	13	21.7
	College	4	6.7
No. of Surviving children (*N* = 60)	0	19	31.7
Mean 1.95 Median 1.00 Mode 0 Range 7	1	14	23.3
	2	9	15.0
	3	5	8.3
	4	5	8.3
	5	2	3.3
	6	2	3.3
	7	4	6.7
Order of pregnancy when fistula occurred (*N* = 60)	1st	26	44.1
	2nd	16	27.1
	3rd	6	10.2
	4th	5	8.5
	5th	3	5.1
	6th	1	1.7
	7th	2	3.4
Total Hospital Visits before surgery (*N* = 60) Mean 4.4 Median 4 Mode 4 Range 8 (1–9)	1	2	3.4
	2	8	13.6
	3	8	13.6
	4	16	27.1
	5	12	20.3
	6	3	5.1
	7	5	8.5
	8	2	3.4
	9	3	5.1
Total No. of Surgeries (*N* = 60) Mean 1.71 Median 1 Mode 1 Range 5	1	34	57.6
	2	11	18.6
	3	6	10.2
	4	3	5.1
	5	4	6.8
	6	1	1.7
Ever had fistula surgery prior to this one? (*N* = 60)	Yes	24	40.7
	No	36	59.3
Where did you deliver the baby? (*N* = 60)	Home	11	18.3
	TBA (Traditional Birth attendant)	2	3.3
	Hospital	47	78.3

Time in years lived with fistula Mean 9.6 Median 4.9 Mode 4.0 Range 38.8 (0.17–39.08)

The research team conducted follow up visits to the participants in their home community setup for 6 and 12 to 19 months after undergoing surgery for obstetric fistula and discharge from hospital. At each visit, in-depth interviews were conducted with the participants.

### Data analysis & development of the theory

We used grounded theory, a qualitative methodology that is flexible, reflexive, incremental and emphasises on patients experiences and interactions. Using a critical realist perspective [15], we take a philosophical standpoint of critical realism and use the classical Glaserian grounded theory methodology. Primary data obtained from using an in-depth interview guide to obtain narratives [16]. Narrative approach allowed participants to construct the events surrounding the index birth and context of occurring fistula illness, living with fistula and their health seeking behaviour. These interviews were audio recorded, transcribed and imported into QSR NVivo 10 software.

Three steps of data analysis namely open coding, axial coding and selective coding was applied on all transcripts [15, 17]. We used an iterative process of data collection, analysis and more data collection analysis until thematic saturation was reached. In this study, theoretical saturation was arrived at the 38th narrative. Conducting narratives at six and after twelve months post discharge allowed for several opportunities to hear the participant’s story as it unfolds and to compare events within the same participant’s story. This technique, referred to as repeated measurement in grounded theory methodology, produced rich and nuanced data for our study. Emergent themes are presented in the results section.

Further analysis developed conceptual frameworks based on the data to explain the theory of reintegration and regaining normalcy for fistula patients. Deviant case analysis took into consideration the untypical narratives of fistula patients, thus included women of varied ages, level of education, geographical location, type of facility and number of children describe all possible conditions of fistula patients across three hospitals in Kenya. Further, we present untypical outcomes of reintegration such as new integration, which is not mentioned in the literature before. We conducted long rigorous fieldwork in an ambitious project, obtaining quality data and generated for the first time to the best of our knowledge, a theory of reintegration and regaining normalcy for obstetric fistula patients.

### Reflexivity and positionality

Our research team composed of a female PhD student who is a nurse with training in social science and two anthropologists as research assistants tried to be as un-intrusive as possible and to dress simply in a way that would not introduce power play. We also assumed a naïve positionality. Nevertheless, in the rural areas where poverty is rife, our research team’s arrival from the city already placed us at a pedestal higher than our rural hosts. All three had met and recruited study participants during the first phase of the study, hence establishing rapport and trust that assisted in the smooth conduct of the second phase of the study. The audio recordings were transcribed by an education expert with skill in transcribing qualitative data. Data collection for this study was not in any way part of or organized together with any scheduled medical follow-up of patients. We held an assumption that surgery improves the lives of women and not much further help is expected from the surgery although this was refuted by the findings.

### Ethics

Ethical approval and appropriate renewals were obtained for the study from the Kenyatta National Hospital and University of Nairobi Ethics and Research Committee (ERC) approval number P618/11/2012. Participants signed a written informed consent form to participate in the study. A consent information sheet was used to inform participants verbally and in writing of the nature of the study, their role in participation, the absence of financial risk nor financial gain from participation and the voluntary nature of participation. Consenting women who participated gave verbal consent of audio recording and concealment of their identity in publishing the results of the study. Participants did not bear any monetary cost to participate in this study, since we visited them in their residence for the interviews.

## Results

This section summarises the key findings of the study objective focusing on the reintegration of fistula patients. These emergent themes resulted in the constructed conceptual framework and theory of regaining normalcy for fistula patients in Kenya. Grounded in this data, participants described reintegration as ‘a state in which the obstetric fistula patient regains optimal physical, psychological and social wellbeing and ‘normalcy’ akin to that of before fistula illness’. In this instance, normalcy was based on the participants’ perspectives on what a return to normal life means to them, the emic perspective. On the other hand, we reveal an emergent category of new integration, describing ‘a state in which a fistula patient starts a new life away from previous social settings, forging new social relations and occupations’. In the theory of regaining normalcy, we argue that an ideal continuum of care of fistula patients should start immediately after symptoms are diagnosed to when the woman resumes normal life as that which they lived before the illness occurred.

As demonstrated in literature and the study findings, there are three distinct thematic phases in the women’s narratives that overlap, interplay and culminate into an outcome as represented in the framework, namely, living with fistula illness, health seeking behaviour, and reintegration and regaining normalcy. The first two phases are contained in separate publications [11, 12] but offer a platform for explaining the final phase covered in this paper. This paper focuses on the reintegration framework for fistula patients’ that is at the tail end of the three thematic phases.

Seeking care is the first step in women’s reintegration and regaining normalcy journey, where they have expectations of resuming their previous normal lives and depends on their perception of ‘normalcy’. Having obtained corrective surgery, patients expect to regain a certain standard of both physical and psychosocial healing as before the illness. In our findings, regaining normalcy is a phrase that best describes the participant’s description of the process of reintegration. It is as though, with the fistula illness, an anomaly, a disorder had occurred to them. Part of their journey after surgery is then spent in seeking that in their life that was normal, before the illness disrupted their lives. We therefore use the term regaining normalcy synonymously with reintegration. The central category that holds our proposed theoretical model together is the reintegration and regaining normalcy process.

### Reintegration and regaining normalcy outcomes

We argue that the varied typologies of reintegration outcomes are a result of the reintegration process; reintegration in our view is both a process along time and a desirable outcome for fistula patients. Our analysis demonstrate that there are four possible outcomes of the reintegration process after fistula surgery, namely reintegrated, partly reintegrated, not reintegrated and newly reintegrating. Basing on the women’s own definition of normalcy, we report results of 32 (53.3%) of the women as reintegrated and a further 4 (3.3%) of the women as newly integrating. There however remain a large proportion of women who are partially reintegrated (33.3%), representing a third of the study participants and small but problematic section of those who are not reintegrated (3.3%).

A typical narrative of participants who were deemed to be reintegrated revealed no remnant symptoms of urine or faecal incontinence, and the physical ailment that resulted from obstetric fistula had been resolved. Mentally, these participants expressed satisfaction with their status and no longer harboured feelings of shame, anger, suicidal thoughts as before surgical treatment. Such participants also had good relationship with the husband, were sexually active without problems and were fully supported by their husband. Reintegrated participants interacted well with her peers and friends, offering her a social platform. They could carry out house chores of cooking, cleaning, fetching water other chores carried out by women in the community. The women were also involved in farming, small business or other forms of earning income for a living. A quote that illustrates a typical narrative of a fistula patient who had regained a normal life after surgery and was ‘reintegrated’ is shown below:

*R:Oh my life after I was treated has gone back to being normal because in the beginning. I had stress but now the stress begun decreasing bit by bit although not that much but then it is not like it was in the beginning. Because you see I was busy concerning myself with business issues, here in the farm that is I can do all that work at once. At times I am at the farm, then at times I am there and there but then in the beginning I couldn’t do any work.*

*I: Okay and is there anything else that has changed since you were discharged?*

*R: Something that has also changed is my social life, you know I had alienated myself for a while and although it was for a short time I had alienated myself, and I can even now go to church because I had stopped going to church. I can go to church, I can visit my friends, I can go to the ‘chamas’[womens table banking groups] very freely without any problem. (020_1F).*

The participants deemed to be ‘not reintegrated’ were having similar challenges to those exhibited when they had fistula illness. They were incontinent of urine and or faecal matter, still experienced shame and were isolated, had been separated or divorced with no support from their husbands. Some harboured suicidal ideation. The continued incontinence was particularly distressful to these participants. These participants were however few. .

Particularly, participant 032_1F who exemplifies this categorisation was having her marriage opposed by her mother in-law because of the fistula illness was severely distressed. Having undergone two previous surgeries with the Rectovaginal Fistula (RVF) being successfully closed, she remained with vesicovaginal fistula and leakage of urine. It did little that she had a surviving child and still lived in her husband’s hut; she was simply still living with fistula illness with the hygiene and social challenges it posed and had to bear the psychological and physical burden of fistula illness. Her anxiety to get successful surgery is captured in the following excerpt:

*Until now the leakage is worsening compared to the other time after operation. I have been so worried of my condition that made me go back to my parent to enquire what I will do about. She suggested that I be taken to Matany hospital in Uganda hopefully that I can get operated since Sentinelles have left. The plan was to go after planting our shamba [April].) I thank God you have come. Hopefully I will get operated. (032_1F*).

For instance, another participant who was not reintegrated explained how she was stressed by her illness and expressed the desire to be normal again:

*It is now like you have the stress of thinking of how you are and how it can be possible to regain your normal status how you used to be earlier. (029_3F).*

Partly reintegrated participants reported improvement in or no symptoms of urine or faecal incontinence. However, they had remnant problems developed due to their previous illness. Some had sexual health problems, were still incontinent, were separated from the husband, felt suicidal, had miscarriages or could not conceive and were struggling with infertility. Others yet were afraid to fall pregnant again. These problems further jeopardised the participants’ marital relationships and their ability to meet societal expectations on them to bear children as women, and often led to poor reintegration outcomes. A few had to adjust to living with complicated fistula surgery like urinary diversion, and had little social interactions. We present excerpts from three women who symbolise the typical ‘partially reintegrated’ category. A woman, who was separated from her husband due to the illness, though now continent, lamented that she couldn’t get back with her husband thus:

*Physically it is normal but then in marriage he has already married. I hope it will go back to normal but then it will be difficult because I personally do not know the one he married how she is. I have seen her but then I do not know how she is. You know nowadays it is bad, nowadays life is difficult. Okay I can go back, we might get back together and then she sees that this one and this one are back together and then she starts having an affair and then we infect each other [HIV]. I am very scared. (018_2F).*

Another participant, though now continent of urine and still together with her husband, was afraid of obtaining another pregnancy, reasoning that it would cause her to develop another vaginal fistula. She remarked:

*You know I even don’t know how I even thought I just began being scared. You know at the hospital they said if you become pregnant again you shall have a caesarean at 8 months; you do not wait until 9 months are over. I now thought what is that now? and again you know in there it is not that good. Even right now my husband doesn’t know that I don’t think that I am going to conceive again. I thought that I better stay like that because what if I go get operated on again and it becomes like the first [delivery] (006_3F 6 months).*

The fertility concerns of recovering fistula patients may be linked to their lack of children, given 31.7% of the participants had no surviving child. A woman who had no surviving child explained her reluctance to obtain RVF surgery because she thought it would prevent her from conceiving. Six months after VVF surgery, the couple are anxious to try for a child and the participant voiced her concerns thus:

*R: It was hard for me […] I thought I might get hurt a lot. I saw that if they had closed the one for urine I would have no problem with that one [RVF] because I can control myself with that one. I thought that that one would wait for the time if God would be graceful to me and I deliver I would then get helped then. Because I thought if I would have that operation [RVF] then it would be hard to get a child. … His concern, he is praying so that the months might end and I may get the strength and may be God will be gracious to us, we get a child. Because he thought maybe when the birth canal was being repaired it may have been affected to cause not giving birth. (031_3F 6 months).*

The last outcome category is that of newly integrating women who though were continent and devoid of other physical symptoms, and were engaged in rebuilding their lives after surgery, they attempted to regain a normal life away from their previous social settings. Having no home to go back to due to divorce or abandonment by family or divorce and separation by the spouse, they moved to new regions, made new friends and started afresh. These participants met a high level of agency in regaining a normal life. For instance, participant 008_1F and 042_2F fully embodied this rare category. Newly integrated participants were socially removed from environments that were previously stigmatising and stressful during fistula illness; they did not depend on anyone for material or economic sustenance and were forming totally new social relations, notably away from maternal or matrimonial home. It is such ‘uncommon’ categories that grounded theory methodology allowed to emanate from this study, which are entirely true of the participants narratives.

The four outcomes of the reintegration process applied to and fit all 60 study participants’ narratives. In our study, there wasn’t any participant whose situation fell out of these four categories described. Further, at different points in time, say at six and nine months follow up, some participants who had been not reintegrated moved along the continuum to partially reintegrated or found new environment to new integration. The reintegration continuum is therefore not static but embodies a mobile and flexible tendency of humans finding a new normal after traumatising and shaming reproductive health illnesses. However, the assumption that women automatically regain normal lives immediately after surgery was refuted in almost half the cases in our study.

### Reintegration process and continuum of regaining normalcy

Reintegration doesn’t occur instantaneously but in a process for most fistula patients following corrective surgery. The process of regaining normal life is wrought with challenges as well as aided by various enabling factors. Thus, women’s reintegration process is dependent on conditions that interact and overlap to determine the reintegration outcome on a continuum of reintegration and regaining normalcy of each participant, which we categorise as ‘enablers’ or ‘disablers’ of reintegration. Thematic analysis yielded seven categories that were deemed to enable a better reintegration outcome for fistula patients, which we call ‘enablers’. Table 2 below shows the full description of enablers and disablers of reintegration.

**Table 2:** Thematic analysis of enablers and disablers of reintegration and regaining normalcy

First order category/ theme	Second order category/ theme	*Category full description*	Third order category/subtheme
Reintegration and regaining normalcy	Positive social capital & interactions	Participants social interactions post fistula surgery. Women who had greater social capital with positive social interactions deemed themselves to be regaining a normal life and previously meaningful social status which they had lost during the illness.	- Being accepted and supported by family - Being accepted and supported by spouse - Being accepted and supported by community
	Continence and physical health	How participants perceive their physical health. Being continent of urine or faecal matter (successful closure of fistula with no leakage of urine or faeces) placed a patient on a new pedestal, removing the hygiene challenges they had before.	- Urine / faecal continence - Perception of physical health - Expectations of surgery
	Performance of gender roles	How a women perform roles expected of them as women in the community. They discussed their roles in relation with their physical condition after surgery, and the coping with the discharge instructions of avoiding manual work. Women who can perform gender roles as expected by society had better outlook towards regaining their normal life after surgery. Gender role insufficiency occurred when women couldn’t fulfil their expected gender roles perceiving themselves as not yet regained normal life.	- Conducting household chores of fecthing water, cooking, sweeping - returning to the farm - Selling goods in the market - Observing discharge instructions to avoid strenous work for 6 months
	Ability to conceive and bear a child	Return to full reproductive function, in terms of being able to give birth and achieve the previously disrupted life goal of motherhood. Includes tension between participants’ desire to give birth soon after surgery (and often the stillbirth during the index birth at fistula) and adhering to discharge instructions of abstinence for 6 months post surgery and postponing conception until after two years.	- Observing discharge instructions to abstain from sexual intercourse for 6 months - Fertility concerns - Perception of and / or using family planning
	Economic independence	Having a form of economic independence, and obtaining a form of skill training was relieving to women who, during the fistula illness, had to rely on relatives for provisions and sustenance during the illness. Dependence for sustenance - Emergent theme in participant’s narratives, of women having to depend on kin and well-wishers for their basic needs after surgery.	- Having own business - Ability to conduct previous income generating activities like farming or selling in the market - Obtaining sewing skills training and machines as part of reintegration support
	Marital stability	Retaining a marriage that was stable with a supportive husband or spouse seemed to bear fruit in psychological and social support, and the possibility of still trying for pregnancy and a child. Women who experience periods of separation from the spouse or even divorce as a result of fistula illness or as a coping mechanism to adhere to discharge instructions of abstinence, experience a negative perception of regaining normal lives.	- Psychological and social support by spouse - Living in marital home - Observing discharge instructions to abstain from sexual intercourse for 6 months - Hope for conceiving again - Divorce and separation
	Mental wellbeing	Participants psychological and emotional thoughts describing suicidal thoughts. Being in a state of mental wellbeing, devoid of stress or anxiety related to fistula illness was beneficial to the process of regaining normalcy.	- Receiving psychological support - Having suicidal thoughts - Mental agony and distress

An example of a dominant emergent category amongst factors disabling reintegration, dependence for sustenance was, is described in this section. Dependence for sustenance explains the helplessness women feel after obstetric fistula surgery. During illness, fistula patients depend on other people especially financially to subsist. This dependence does not end at surgery, markedly increasing because of three main reasons. First, the challenges the surgery poses to the women’s physical health causes inability to perform chores that would bring income. Secondly, they are asked not to perform strenuous work after discharge, and are obliged to adhere to these instructions for better healing. Third, women desire to perform their normal gender roles as expected of women in their communities like cooking, fetching water and firewood cannot be fulfilled and they have to depend on others for this. The overarching helpless feeling makes the women to consider themselves as not reintegrated, since much of what they could do for themselves they now have to depend on someone else for, a state far from the usual or the norm.

**Fig. 1:**
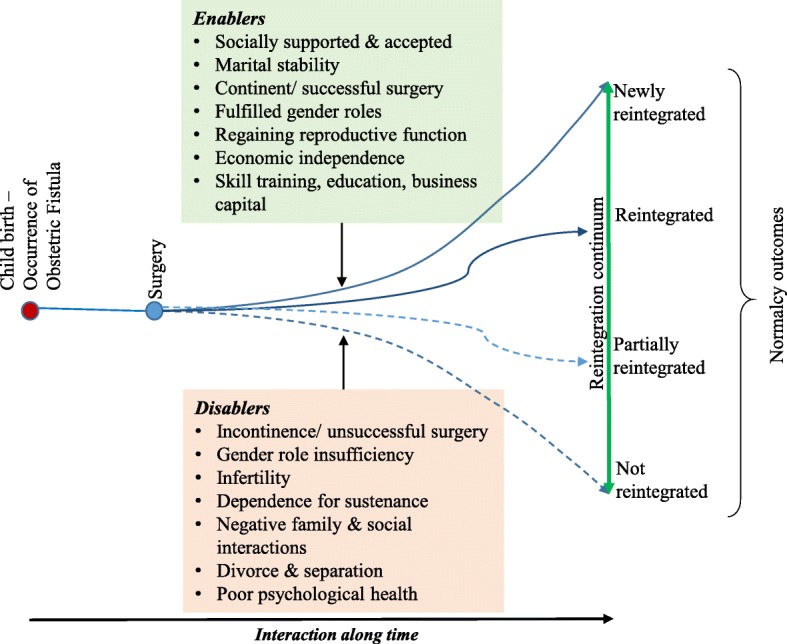
Conceptual framework of reintegration and regaining normalcy for fistula patients

The intersection of the circles of enablers and disablers mean that the patient has both factors for and against their reintegration and regaining normalcy state. They straddle a grey area which is likely to be overlooked in planning to cater for the needs of fistula patients post-surgery. For a fistula patient who is straddled between enablers and disablers, partial reintegration or not reintegrated is a likely outcome on the continuum. The relationship between enablers and disablers of reintegration, consequent outcomes of the reintegration process over time, and the basic tenets of the conceptual model of reintegration and regaining normalcy of fistula patients is illustrated in Fig. 1 below:

Our study reported verbatim the women’s experiences of reintegration, without performing physical examination to determine the continence status of women. Our model does not refute nor emphasize the place of continence in reintegration; there were partially reintegrated women who were continent and others partly continent.

### Towards a grand theory of regaining normalcy: Theoretical tenets

The onset of fistula illness introduces hygiene, social and moral in an illness with chronic trajectory [12]. It imposes a disruption on women’s social life and indents physical and psychological wellbeing. The women then strive to ‘regain normalcy’. Normalcy is defined by the woman’s expectations of the treatment and reintegration process, and is what she terms as having gone back to their normal life as before fistula illness – in terms of social roles, gender roles, participating in education, business and farming, physical health, reproduction, and self-sustenance. These emergent markers of normalcy presented by the women are vital in informing care of fistula patients and are suggestive of a holistic approach to management to ensure reintegration. Whilst each woman had an experience unique to them, the substantive theory proposed herein speaks to and fits the data for all women. The model thus is transferable to fistula patients in a similar context.

Interaction with enablers and disablers over time result in reintegration and normalcy outcomes. Overall, the conceptual framework is best understood by viewing reintegration and regaining normalcy as a process that begins with the occurrence of an illness at childbirth and ends with any of the four reintegration outcomes over a period of time that varies, depending on the individual woman’s contextual factors, from a few months to decades.

## Discussion

Living with fistula illness poses a crisis to women’s lives, introducing a chronic illness that disrupts their normal lives. The characterisation of obstetric fistula as a chronic illness has been suggested by several authors [12, 4, 18]. The length of the care process, limitations of surgery necessitating repeat surgeries and the persistent physical and moral suffering due to fistula illness qualify the disease to be considered as chronic [4]. Similarly, the long period of months to decades living with fistula illness in the health seeking pathways make the illness to take on a chronic trajectory [12]. Often, patients perceive themselves as suffering from a chronic disease that needs long term care [4]. Their recovery thus should be analysed and discussed against the perception of obstetric fistula as an illness the follows a chronic trajectory.

We sought to establish the process of healing, reintegration and regaining normalcy for fistula patients in post-surgery period. To the best of our knowledge, no study had attempted to document philosophical underpinnings driving fistula illness care and reintegration outcomes in Kenya nor developed a reintegration theory grounded in data prior to our study.

Available literature on the reintegration of patients with obstetric fistula is limited in scope,, more so that which offers qualitative data, with which to discuss our study findings [19]. However, there has been a growing interest in post repair condition of fistula patients. For instance, in sync with our findings on the centrality of fertility in reintegration of fistula patients, sexual and reproductive health post-repair is often mentioned as a criteria to reduce stigma and smooth reintegration process [9, 20]. One study in Uganda has set out to focus on the complete realm of reintegration outcomes, highlighting the scarcity of data on what a reintegration model would look like for fistula patients in Sub-Saharan Africa. Nevertheless, they identified identifying similar reintegration needs for patients such as social engagement [11].

The findings of our research confirm what other authors have predicted about the healing process and the reintegration of fistula patients [11, 6, 21]. Previous exploratory research on a small section of fistula patients in Kenya identified the presence of family and community support to the patient after surgery as being important in their reintegration, in addition to counselling, provision of follow up care and having income generating activities or skill training for the same [6].

At the end of our study, one comparable study that was a 12 month follow up project of a cohort of 60 women was ongoing to measure reintegration for women who have undergone corrective surgery for obstetric fistula in Uganda [10]. In the initial qualitative phase, the study developed and validated a tool to measure reintegration of fistula patients [11]. The resulting reintegration tool of the study published by [11] identifying four key measures of the concept of reintegration, namely, mobility and social engagement, meeting family needs, comfort with relationships and general life satisfaction.

Our study findings are similar to [11] findings in three ways. Firstly, we found that engaging in social interactions as before surgery was important to women in the recovery period. Secondly, women in our study deemed to have regained their normal lives if they were accepted by and had support from the family and community members. Thirdly, women in our study also perceived themselves to have regained a normal life if they were able to perform their gender roles as expected of women in the home, including conducting domestic chores. However, contrary to our study, the [11] study did not include resumption of normal sex life as a reintegration concern for women after fistula surgery, as opposed to our findings which emphasised on women being concerned with resuming their sexual and reproductive or fertility capabilities as before the illness. Conversely, [11] study is silent on remnant urine or faecal incontinence, which often was a frequent concern amongst our study participants.

On the other hand, [22] reported that women realised improvements in social life, depending on the outcomes of the surgery, with those still incontinent being less likely to be living with their husband at 3 months post-surgery [22]. These findings resonate with our study findings. It would be interesting to sample the findings of the Ugandan study, especially the phase investigating reintegration outcomes as measured by their validated reintegration tool [10]. Of concern is to compare our identified four reintegration outcomes to those of patients in the Uganda study.

Similar to our study findings, a systematic review of qualitative studies posited that fulfilment of social roles is the most important factor for women’s rehabilitation following fistula surgery [19]. We found that social interactions were an important factor in the reintegration of fistula patients, with those who had no social support either being not reintegrated as an outcome, or seeking new integration in new social spaces that would accommodate them, mostly in environments where people are not aware of the woman’s previous illness.

One study on the contrary challenges our findings and general suggestions on reintegration [18]. Whilst the [18] study is quite important in challenging the popular image of a fistula patient as one who is an outcast and a social pariah, it does not suggest how obstetric patients should be helped to navigate the reintegration process after surgery, or if at all for these patients, reintegration is necessary. The authors did not comment on the fate of patients after fistula surgery.

Public health studies on fistula illness in Kenya have more often utilised quantitative and mixed methods research [23–25], giving great attention to predisposing factors [23–25]. Other studies, including qualitative studies, have reported on fistula care outcomes [5, 24] psychological illness in fistula patients [23]. Globally, existing literature on reintegration of fistula patients is somewhat limited in scope, with existing studies focusing on continence [8, 26] and management of pregnancy [26] and recurrence of fistula [9] after surgery. There are fewer studies on the holistic quality of life of fistula patients after surgery, though exploratory studies have pointed to a gap in the care of patients during the period after fistula surgery [6, 20]. We thus settled on grounded theory, a sufficiently rigorous qualitative methodology and findings informed the development of this reintegration model framework that is grounded in the data. However, the model was not tested empirically and remains thus transferable to fistula patients in similar context(s) in Kenya.

A quasi-experimental study pilot testing the suggested interventions would be a welcome development in fistula research.We also suggest other further research that was out of scope for the current study. Basing on our data, there was a progression to better states or to worse states of reintegration especially for partly reintegrated women. Our data was however not sufficient to test the influence of a participants’ past experiences such as time lived with illness and other factors on their reintegration. Whilst we acknowledge the limited scope of our study, we recommend that this should be studied further to enrich our understanding of the influence of the illness experience on reintegration outcomes for fistula patients.

## Conclusion

Women do encounter many problems after fistula surgery and could do with psychological, biomedical, social and economic support. They are a special group needing fertility treatment and specialised follow up during subsequent pregnancy and childbirth that is ultimately expected to be through caesarean section. The assumption that women automatically regain normal lives immediately after surgery was refuted in almost half the cases in our study.

A comprehensive framework for treating fistula patients should focus on prompt diagnosis and improving health seeking behaviour of patients, shortening the length of time and number of points of contact that patients with fistula deal with before obtaining treatment. In addition, after fistula surgery, one cannot assume that the woman will automatically regain their normal lives as before fistula illness. Interventions must be put in place to obtain better outcomes at regaining normal lives and eliminating negative outcomes of partial reintegration or failure to reintegrate. Our framework thus can be used to better understand the points of care that should be instituted to reintegrate fistula patients, thus offering holistic care to fistula patients. We call for further research to test the model proposed in this reintegration framework.

## Abbreviations

ERC: Ethics and Research Committee
RVF: Recto Vaginal Fistula
VVF: Vesico Vaginal Fistula
